# Interleukin-1- Receptor Kinase 4 Inhibition: Achieving Immunomodulatory Synergy to Mitigate the Impact of COVID-19

**DOI:** 10.3389/fimmu.2021.693085

**Published:** 2021-06-23

**Authors:** Akash Gupta, Hyung J. Chun

**Affiliations:** ^1^ Department of Internal Medicine, Yale School of Medicine, New Haven, CT, United States; ^2^ Yale Cardiovascular Research Center, Section of Cardiovascular Medicine, Department of Internal Medicine, Yale School of Medicine, New Haven, CT, United States

**Keywords:** COVID-19, irak4, immunomodulator, cytokine, Cytokine release syndrome (CRS)

## Introduction

COVID-19 is a viral disease caused by a novel coronavirus, SARS-CoV-2, and is responsible for a pandemic since being identified in January 2020, resulting in a severe acute respiratory syndrome (SARS). Although most patients with COVID-19 may experience an asymptomatic, mild, or self-limited illness, many patients rapidly develop dyspnea and pneumonia, requiring hospitalization for pulmonary support. Patients with severe COVID-19 symptoms manifest cytokine release syndrome (CRS), which is associated with systemic inflammation, hemodynamic instability, and multi-organ failure (1). Progression from milder respiratory symptoms to acute respiratory distress syndrome (ARDS) is currently believed to be driven largely due to CRS and be one of main reasons for COVID-19 mortality.

## Cytokine Release Syndrome in COVID-19, Innate Immunity, and Antibody Response

The CRS phase of SARS-CoV-2 is thought to occur due to an influx of neutrophils and macrophages as well as elevations of inflammatory cytokines, with higher levels of IL-6, IL-1, IL-8, and IL-18 (1, 2). In CRS, a variety of pro-inflammatory cytokines, including IL-1, IL-6, IL-8, CXCL-10, interferon (INF)-induced chemokines, and tumor necrosis factor (TNF)-α are secreted by alveolar macrophages that drive the inflammatory response and promote further influx of neutrophils, monocytes, and other inflammatory cells (1, 3).

During the later stages of COVID-19, there are increases in pro-inflammatory cytokines and low levels of antiviral antibodies and adaptive immune responses noted, similar to previously reported results about SARS-CoV, suggesting innate immunity rather than the adaptive immunity as the driving force for excessive inflammation in COVID-19 associated ARDS (3, 4). In Meizlish et al., proteomic profiling of hospitalized patients with COVID-19 revealed prominent signatures of neutrophil activation in those patients with critical illness (5). Markers of neutrophilic activation, such as granulocyte colony-stimulating factor [G-CSF] and interleukin-8 [IL-8] and neutrophil-derived effectors (resistin [RETN], lipocalin-2 [LCN2]) had the greatest discriminatory power in this study for identifying patients with severe COVID-19 (5). This study represented important findings given that neutrophils are often the first responders in the innate immune response but can have notable collateral damage.

Complement activation has also been suggested to play an important role and be a distinct entity in severe COVID-19 infection. In Ma et al., circulating markers of complement activation were found to be higher in patients with respiratory failure in COVID-19 compared to those with non-respiratory failure with COVID-19 and influenza (6). The complement pathway can typically be activated by 3 arms: classical pathway, alternative pathway, and lectin pathway. In this study, increased activation of the alterative pathway was noted and was found to be associated with worse outcomes in COVID-19 infection (6).

Studies are currently ongoing to shed more light on the adaptive immunity and antibody response associated with COVID-19. There is data that demonstrates the ability of the SARS-CoV-2 virus to elicit neutralizing antibody response in sera and for those antibodies to be isolated (7, 8). There is evidence that the antibody response is largely mounted against the spike and nucleocapsid proteins of the SARS-CoV-2 virus and the severity of infection is associated with an increase in magnitude and breadth of the humoral response (9). The kinetics of the humoral response equally important, in addition to the magnitude of it, as delayed production of neutralizing antibodies has also been showed to be linked to fatality and impaired viral clearance (10).

## COVID-19 and Toll-Like Receptors

Activation of human innate immune cells, such as macrophages, through binding of viral antigens from SARS-CoV-2 to cell-surface toll-like receptors (TLRs) has been demonstrated to be a vital mediator of COVID-19 immunopathogenesis (11–13). TLRs are a family of 10 transmembrane receptor proteins (TLR1-TLR10) that recognize pathogen-associated molecular patterns (PAMPs) on viruses, bacteria, and other foreign molecules. TLRs play a major role in the initiation of the innate immune response, with the production of inflammatory cytokines, type I IFN, and other mediators (11–13).

The SARS-CoV-2 viral spike protein binds to the extracellular domains of various TLRs, with strongest binding to TLR4. It has been shown that pathogenic human coronaviruses induce oxidized phospholipids that promote acute lung injury by increase lung macrophage cytokine/chemokine production *via* TLR4 (12). Similarly, SARS-CoV specific GU rich ssRNA fragments induce a high level of TNF- α, IL-6, and IL-12 *via* TLR7 and TLR8 (14). Collectively, recent *in vitro* and *in vivo* experiments suggest that TLRs and innate immunity pro-inflammatory signaling may be important in CRS and major immunopathologic consequences.

## Immunomodulatory Therapies in COVID-19

Immunomodulatory agents, which are commonly used in rheumatologic conditions, have garnered interest for COVID-19 and the hyperinflammatory state. Certain agents are being used to target individual mediators of the inflammatory pathway to mitigate CRS; for example, tocilizumab, which is a monoclonal antibody against the receptor for IL-6, has shown some potential benefit for improving lung function and decreasing length of hospitalization in a large single-center trial (15, 16). Other studies have shown conflicting results with little to no benefit for tocilizumab in mortality of patients with COVID-19 (15, 17–19).

In addition, studies have investigated the benefit of steroid use in COVID-19 patients, given their ability for broad-based immunosuppression. In a trial from University of Oxford including 6000 patients with COVID-19 taking 6mg dexamethasone daily, there was lower mortality in ventilated patients and those on oxygen therapy, with on overall decrease in 28-day all cause mortality (15). However, the use of steroids has only been indicated in a subset of COVID-19 patients with hypoxia and has not been shown to have utility in other aspects of the disease, while carrying the risk of multiple side effects (15).

Targeting multiple cytokines with an agent may represent a viable strategy in the treatment of CRS in COVID-19. Janus kinase (JAK) inhibitors, which target JAK1, JAK2, JAK3, affect multiple cytokines involved in antiviral responses such as type I interferon, IL-2, IL-15, IL-21, and IFNγ (19). Baricitinib, a selective inhibitor for JAK1 and JAK2 commonly used for rheumatoid arthritis, has shown in combination with an antiviral, known as remdesivir, to decrease recovery time for patients with COVID-19, particularly those on high flow oxygen (19). However, ruxolitinib, another selective JAK1 and JAK2 inhibitor, did not show any improvement in recovery time in patients with COVID-19 compared to placebo group (19). Currently, there are limited therapeutic strategies for COVID-19, which may be only used in a subset of patients and provide a modest benefit in recovery times and mortality.

## IRAK4 Axis and COVID-19

**Figure 1:**
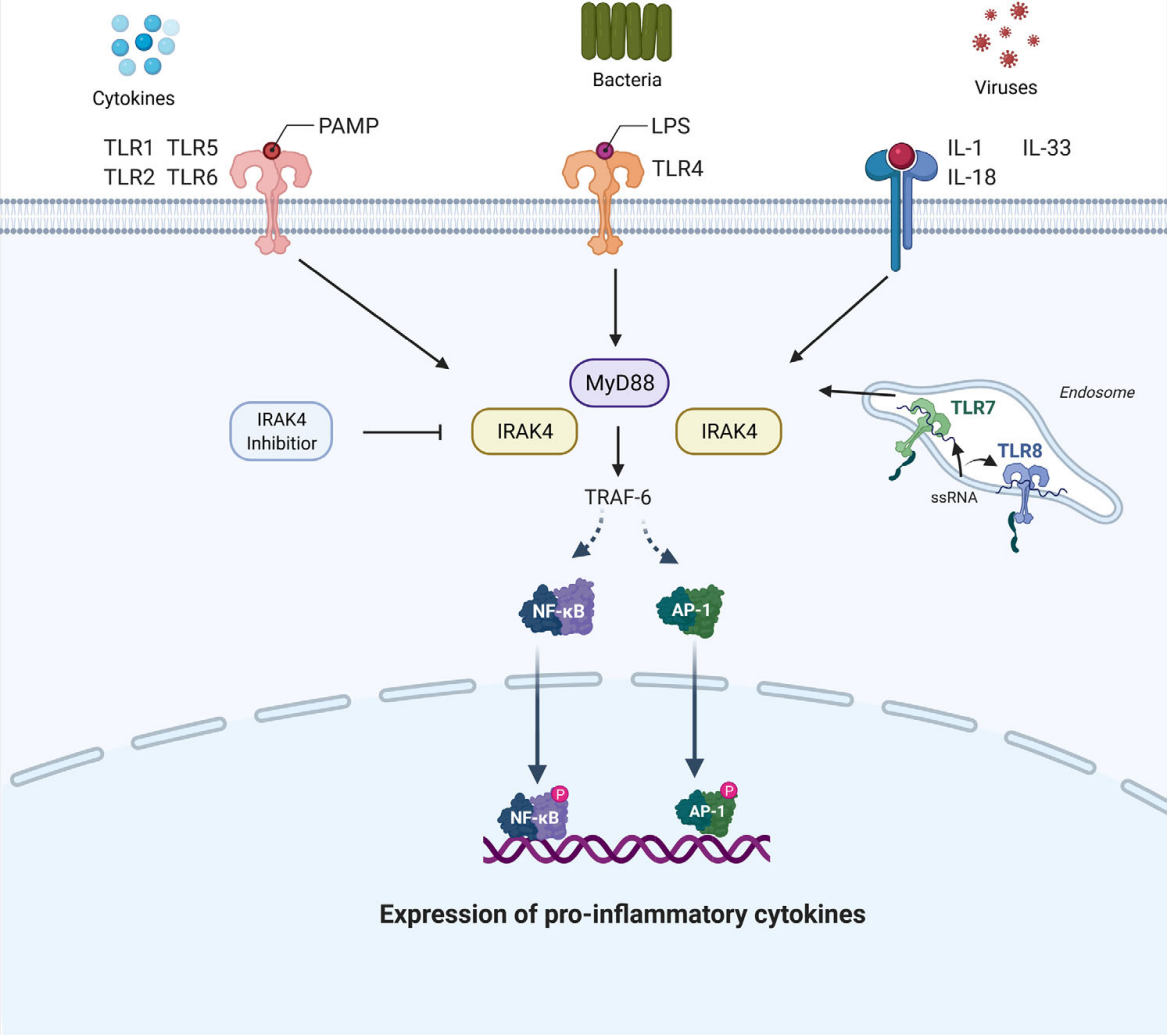
Signaling pathway involving interleukin 1 associated receptor kinase 4 (IRAK4). IRAK-4 is a serine, threonine kinase that is a key intracellular signaling node downstream of myddosome-associated toll-like receptors (TLRs), which is depicted as myeloid differentiation primary response 88 (MyD88), and the IL-1 family receptors (IL-1R, IL-18R and IL-33R). Activation of the IRAK4 pathway triggers an inflammatory chemokine and cytokine cascade that is important in innate immunity. This is mediated through the recruitment and activation of Tumor Necrosis Factor (TNF) receptor associated factor 6 (TRAF6). The TRAF6 adaptor protein is able to interact and induce the translocation of transcription factor nuclear factor kappa B (NF-kB) to the nucleus, resulting in transcriptional activation of genes encoding cytokines and chemokines. Additionally, TRAF6 can induce a pathway through mitogen-activation protein kinases (MAPK) that leads to activator protein 1 (AP-1)- induced gene expression of pro-inflammatory cytokines. In addition, ssRNA fragments from SARS-CoV-2 virus can activate the IRAK4 pathway as shown *via* TLR7 and TLR8, which are membrane bound on an endosome, as shown. As shown, an IRAK-4 inhibitor will inhibit this inflammatory chemokine and cytokine cascade. This pathway can be activated by other cytokines or by the recognition of TLRs with pathogen-associated molecular patterns (PAMPs) on viruses, bacteria (e.g. lipopolysaccharide (LPS) on gram negative bacteria and TLR4), and other foreign molecules.

The cytokines that are correlated with poor prognosis in ARDS (IL-6, TNF and IL-1) are controlled by the TLR-interleukin 1 associated receptor kinase 4 [IRAK4]-interferon regulatory factor 5 [IRF5] axis (20). IRAK-4 is a serine, threonine kinase that is a key intracellular signaling node downstream of myddosome-associated TLRs and the IL-1 family receptors (IL-1R, IL-18R and IL-33R) that mediate much of the human innate immune responses (**Figure 1**). In mice with genetically deleted IRAK4, the TLR/IL-1 signaling is impaired, resulting in limited proinflammatory cytokine profile (20, 21).

Inhibition of IRAK4 kinase activity blocks the production of cytokines, such as type I IFNs, inflammatory cytokines IL-6, TNF-α, IL-12 and IL-1, which are key drivers in the pathogenesis of multiple autoimmune and inflammatory diseases. In mouse models, it has been shown that IRAK4 inhibitors suppress lipopolysaccharide-induced TNF activation, alleviate collagen-induced arthritis, and block gout formation (21). IRAK4 has thus emerged as an attractive therapeutic target for diseases associated with dysregulated inflammation, such as systemic lupus erythematosus (SLE), rheumatoid arthritis (RA), spondylarthritis, and psoriatic arthritis. In a phase 2b, multi-center, double-blind, randomized clinical trial for patients with RA inadequately treated with methotrexate, patients taking a reversible IRAK4 inhibitor had a greater decrease in clinical disease scores and inflammatory markers compared to the placebo group (22). Ongoing clinical trial (NCT04575610) is investigating the efficacy of IRAK4 inhibition in patients who are hospitalized with COVID-19 ARDS.

At the present, studies have been focused on targets in the hyperinflammatory state associated with SARS-CoV-2 and improving disease outcomes. Some cytokine inhibition strategies are being investigated for COVID-19; however, IRAK4 may thus provide an important therapeutic target to consider for CRS in COVID-19, given its role in innate immunity and TLR signaling. The potential impact of a successful therapeutic target, such as IRAK4, in COVID-19 could allow for decreased overall mortality, reduced time with mechanical ventilation, decreased time to clinical improvement, and shorter hospitalization stay.

## Author Contributions

All authors listed have made a substantial, direct, and intellectual contribution to the work, and approved it for publication.

## Conflict of Interest

The authors declare that the research was conducted in the absence of any commercial or financial relationships that could be construed as a potential conflict of interest.
